# Heart rate variability during sleep in synucleinopathies: a review

**DOI:** 10.3389/fneur.2023.1323454

**Published:** 2024-01-04

**Authors:** Adeel A. Memon, Ethan B. George, Talha Nazir, Yatharth Sunkara, Corina Catiul, Amy W. Amara

**Affiliations:** ^1^Department of Neurology, West Virginia University Rockefeller Neuroscience Institute, Morgantown, WV, United States; ^2^Department of Neurology, University of Alabama at Birmingham, Birmingham, AL, United States; ^3^NeuroCare.AI, Neuroscience Academy, Dallas, TX, United States; ^4^Department of Neurology, University of Colorado, Anschutz Medical Campus, Aurora, CO, United States

**Keywords:** heart rate variability (HRV), synucleinopathies, Parkinson's disease, sleep, Alzheimer's disease, Lewy body dementia, multiple system atrophy, rapid-eye-movement sleep behavior disorder (RBD)

## Abstract

Synucleinopathies are a group of neurodegenerative diseases characterized by abnormal accumulations of insoluble alpha-synuclein in neurons or glial cells. These consist of Parkinson's disease (PD), dementia with Lewy bodies (DLB), and multiple system atrophy (MSA). Moreover, idiopathic REM sleep behavior disorder (iRBD) is often the first manifestation of synucleinopathies, demonstrating a pathophysiological continuum. While these disorders vary in prevalence, symptom patterns, and severity, they can all include autonomic nervous system (ANS) dysfunction, which significantly reduces quality of life and worsens prognosis. Consequently, identifying abnormalities of the ANS can provide opportunities for improving quality of life through symptomatic treatments that are tailored to the individual's symptoms. An exciting development is using heart rate variability (HRV) as a non-invasive research tool for analyzing how the ANS regulates physiological processes. HRV during sleep, however, may provide a more accurate and reliable measure of ANS activity than during wakefulness, as during awake time, ANS activity is influenced by a variety of factors, including physical activity, stress, and emotions, which may mask or confound the underlying patterns of ANS activity. This review aims to provide an overview of the current knowledge regarding sleep-related HRV in synucleinopathies and to discuss contributing mechanisms. Evidence suggests that iRBD, PD, and MSA are associated with nocturnal ANS dysfunction. Further, comparative studies indicate that the presence of RBD could exacerbate this abnormality. In contrast, no studies have been conducted in patients with DLB. Overall, this review provides new insight into the complex interplay between the ANS and synucleinopathies and underscores the need for further research in this area to develop effective therapies to improve sleep and overall quality of life in patients with synucleinopathies.

## Introduction

Synucleinopathies are a group of neurodegenerative disorders characterized by abnormal aggregation of alpha-synuclein protein in the brain. These conditions include Parkinson's disease (PD), multiple system atrophy (MSA), and dementia with Lewy bodies (DLB). Rapid eye movement (REM) sleep behavior disorder (RBD) is an early manifestation of synucleinopathies (1–4). Sleep disorders including RBD are common in synucleinopathies, and can significantly impact quality of life (5, 6). In PD, RBD prevalence ranges from 35% to 50%, while in MSA it can be as high as 90% (7–9). Currently, however, there is no evidence that juvenile-onset RBD, such as would be associated with narcolepsy, is associated with synucleinopathies. Due to the lack of non-invasive biomarkers, there are no good treatment options, resulting in worsened quality of life (5).

During sleep, heart rate variability (HRV) serves as an objective, non-invasive assessment of autonomic nervous system (ANS) function in people with neurodegenerative disorders (10). HRV reflects beat-to-beat heart rate variation and is regulated by the ANS. Sleep stages and circadian rhythms influence HRV. For example, the parasympathetic nervous system (PNS) dominates during non-REM (NREM) sleep, particularly during deep sleep (slow-wave sleep), resulting in increased HRV (11). Conversely, during REM sleep, the sympathetic nervous system dominates, reducing HRV (12). Furthermore, circadian factors influence HRV, with HRV being highest during the nighttime and lowest during the daytime (13). Synucleinopathies reduce HRV during sleep, possibly related to the degeneration of brain regions controlling the ANS (14, 15). Thus, HRV analysis during sleep may provide insights into potential biomarkers, therapeutic interventions, underlying mechanisms of autonomic dysfunction, and early warning signs for disease progression and complications.

Sleep-related heart rate variability (HRV) can be analyzed in several ways, including time-domain, frequency-domain, and non-linear analyses (14). Time-domain analysis measures the variation in intervals between successive heartbeats. Commonly used time-domain measures of HRV during sleep, which provide general information about ANS activity, are the standard deviation of normal-to-normal (NN) intervals (SDNN), root mean square of successive differences (RMSSD), and percent of NN intervals that differ by more than 50 ms (pNN50). Frequency-domain analysis uses Fourier transform to convert time-domain signals into frequency components. The three standard frequency-domain measures of HRV during sleep are low-frequency power (LF; 0.04–0.15 Hz, proposed to represent combined sympathetic and parasympathetic influences and baroreflex modulation), high-frequency power (HF; 0.15–0.4 Hz, proposed to represent parasympathetic influences), and the ratio of LF to HF, which is thought to represent sympathovagal balance (16). Table 1 describes these and additional HRV measures. Non-linear analysis measures the complexity of heart rate signals using complex mathematical models. Non-linear measures of HRV during sleep include approximate entropy (ApEn), sample entropy (SampEn), and detrended fluctuation analysis (DFA). Despite non-linear analyses providing new insights into HRV, applications to clinical outcomes are unclear and therefore this analysis will not be discussed further in this review. Rather, this review will summarize available literature exploring HRV during sleep in patients with synucleinopathies including RBD (Table 2).

**Table 1:** Heart rate variability measures and abbreviations.

**Metric (Abbreviation)**	**Relevance**
**Temporal domain analysis**	
RR Interval (RR)	Interval between successive R waves in the QRS complex measured in time
RR50	Represents the number of subsequent RR intervals that differ by more than 50 ms
Normal-normal interval (NN)	Interval between normal/normalized successive R waves in the QRS complex measured in time
Standard deviation of NN intervals (SDNN)	The standard deviation of NN intervals during a period of interest
Standard deviation of the average NN intervals (SDANN)	The standard deviation of the averages of NN intervals during 5 min segments of the entire recording
Number of adjacent NN intervals differing > 50 ms from previous (NN50)	Requiring a 2 min epoch, NN50 notes the number of adjacent NN intervals that differ from each other >50 ms
Percent of NN intervals differing > 50 ms from previous (pNN50)	Requiring a 2 min epoch, pNN50 notes the percentage of adjacent NN intervals that differ from each other >50 ms
Root mean square of successive differences of NN intervals for period of interest (RMSSD)	Reflecting beat-to-beat variance in heart rate, RMSSD is the root mean square of successive differences between normal heartbeats.
**Frequency domain analysis**	
High frequency power (HF)	HF band (0.15–0.40 Hz) reflects parasympathetic activity
High frequency power normalized units (HFnu)	HF power normalized to total power
Low frequency power (LF)	LF band (0.04–0.15 Hz) reflects both sympathetic and parasympathetic activity
Low frequency power normalized units (LFnu)	LF power normalized to total power
Very low frequency power (VLF)	VLF band (0.0033–0.04 Hz) reflects parasympathetic activity and denotes non-autonomic renin-angiotensin system effects
Ultra-low frequency power (ULF)	ULF band (≤ 0.0033 Hz) implicated in very slow-acting biological processes
Ratio of LF to HF (LF/HF)	LF/HF ratio is associated with sympathovagal balance. A low LF/HF ratio reflects parasympathetic predominance; a high LF/HF ratio reflects sympathetic predominance.
Total power (TP)	Reflects total HRV through variance of all NN intervals. TP is the sum of energies in the ULF, VLF, LF, and HF bands during a 24 h period and the VLF, LF, and HF bands for short-term recordings.

**Table 2:** Available literature investigating heart rate variability in synucleinopathies.

**References**	**Country**	**Disease**	**N**	**Study design**	**Methods**	**Results/Findings**
Abele et al. (17)	Germany	SAOCA and MSA	Sleep study: 7 MSA 3 SAOCA 5 Controls Wakefulness study: 17 MSA 17 SAOCA 17 Controls	Case-control	• Analyzed standard 8-h PSG in sleep for two consecutive nights • Analyzed 300 second body-movement-free periods from stages: NREM2, NREM3/4, REM, and wakefulness prior to sleep • Frequency domain values computed: HF, LF, HF/LF	• Significantly increased HFnu in MSA compared with SAOCA during-REM sleep and compared with controls while awake, during REM-sleep, and during NREM3/4.
Bugalho et al. (18)	Portugal	iRBD and PD	10 iRBD 18 PD with RBD 8 PD without RBD	Comparative	• Analyzed 5-min epochs selected from wake, NREM, and REM • Time domain values computed: NN, SDNN, and RMSSD • Frequency domain values computed: LF and HF	• No HRV differences found between PD and iRBD groups. • Lower HF values found in patients with IRBD compared to patients without iRBD
Brisinda et al. (19)	Italy	PD, MSA	23 PD 13 MSA 40 Controls	Case-control	• Frequency domain values computed: LF, HF, and LF/HF ratio	• Both PD and MSA patients had significantly lower LF and HF during sleep and wakefulness
Covassin et al. (20)	USA	PD	18 PD with RBD	Retrospective	• Analyzed 2 min from every sleep stage • Frequency domain values computed: LF, HF, and LF/HF ratio • HRV measures were averaged for NREM and REM sleep periods. Average overnight values were also calculated.	• Significant negative relationships between the HF during REM and the H&Y Score and UPDRS total score • Overnight LF/HF ratio was positively related to the H&Y staging, UPDRS total score, rigidity, and hypokinesia
Dijkstra et al. (21)	Belgium	iRSWA	49 iRSWA 41 Controls	Case-control	• Analyzed 5-min epochs of ECG data during wake (at rest), N2, and REM • Time domain values computed: NN and SDNN • Frequency domain values computed: VLF, LF, HF, and LF/HF ratio	• Median NN intervals were smaller in the iRSWA group than in the control group during all stages of sleep, but the difference was not statistically significant.
Ferini-Strambi and Smirne (22)	Italy	PD, AD, MS, and RBD	26 PD 16 AD 25 MSA 14 RBD 15 Controls	Case-control	• Standard 8-h PSG from two consecutive nights. • Sleep stages scored according to Rechtschaffen and Kales • Evaluated RR interval., R_s/w_, R_bm_	• More than one third of patients with presenile AD had defective cardiac control. • Untreated PD patients showed predominantly defective parasympathetic (decrease R_s/w_), and to a lesser extent sympathetic (decrease R_bm_), function during sleep.
Haapaniemi et al. (23)	Finland	PD	54 Untreated PD 47 Controls	Case-control	• HRV analysis conducted using 24-h ambulatory ECG • Frequency domain values computed: LF, VLF, and HF	• Patients with PD had significantly lower SDNN, VLF, LF, and HF values compared to controls. • Patients with mild hypokinesia had higher HF values than patients with more severe hypokinesia
Kitae et al. (24)	Japan	MSA	7 MSA 7 Controls	Case-control	• Time domain value computed: RR50 • Frequency domain values computed: LF, HF power (measured every 5 min) • Differences in the averages between SBP, DBP, PR, HR, RR50, LF and HF between waking and sleeping periods were also computed.	• Compared to controls, MSA patients had lower RR50, LF, and HF values during sleep • Controls had significantly lower LF/HF ratio during sleep than during the waking period, but patients with MSA did not
Kasanuki et al. (25)	Japan	DLB, AD	30 Probable DLB 30 Probable AD 20 Controls	Case-control	• HRV determined through 5-min EEG recording of RR intervals. • Time domain values computed: NN, SDNN, pNN50, and RMSSD • Frequency domain values computed: VLF, LF, HF, and total spectral power	• DLB group showed significant decreases compared to AD group in almost all HRV parameters including SDNN, pNN50, RMSSD, VLF, LF, HF, and total power.
Lanfranchi et al. (26)	Canada	iRBD	10 iRBD 10 Controls	Case-control	• Analyzed 5-min segments from stages NREM, and REM • Time domain values computed: NN, SDNN, and pNN50 • Frequency domain values computed: LF, HF, total power, and LF/HF ratio	• HF, and HFnu components decreased from NREM to REM in controls but did not change in RBD subjects • LFnu and LF/HF increased from NREM to REM sleep in controls but remained stable in RBD subjects.
Mastrocola et al. (27)	Italy	PD	13 PD 13 Controls	Case-control	• HRV analysis conducted using a continuous 24-h ECG • Time domain value computed: mean SDNN intervals • Frequency domain values computed: LF and HF	• PD patients had reduced SDNN and LF during the full 24-h period compared to controls • PD patients had reduced SDNN, LF, and HF during the night compared to controls
Niwa et al. (28)	Japan	PD	27 PD 30 Controls	Case-control	• HRV analysis conducted using 24-h ambulatory ECG • Frequency domain values computed: total frequency LF, HF, and LF/HF ratio	• Total frequency component and LF/HF ratio were lower in PD patients • Compared to controls, PD patients had lower HF in bed, but higher HF out of bed
Palma et al. (16)	Spain	PD	33 PD 29 Controls	Case-control	• Analyzed 10-min transformed and averaged epochs throughout sleep periods: REM, N1–N2, N3, wakefulness before sleep (W-pre), and wakefulness after sleep (W-post). • Time domain values computed: NN and SDNN • Frequency domain values computed: ULF, VLF, LF, HF, and LF/HF	• Mean RR intervals were significantly lower in PD patients than control subjects in all sleep stages, except in N1–N2. • There were no significant differences in the SDNN parameter in any sleep stage. • For PD Patients: ULF was lower during N3, VLF and LF were lower during REM, and HF was lower during N1–N2 • In PD patients, UDPRS-ON and UPDRS-OFF were inversely correlated with VLF and LF during REM
Pyatigorskaya et al. (29)	France	PD	52 PD 24 Controls	Case-control	• HRV analysis conducted using continuous overnight ECG monitoring • Frequency domain values computed: LF, LFnu, HF, and HFnu	• PD patients had significantly lower LF and higher HF compared to controls during REM sleep.
Pursiainen et al. (30)	Finland	PD	44 PD 43 Controls	Case-control	• Time domain values computed: SD intervals • Frequency domain values computed: LF and HF	• PD patients had reduced LF and HF during the night compared to controls • The night-to-day ratios of HRV measures did not differ significantly between patients and controls.
Sauvageot et al. (31)	Luxembourg	PD	35 PD 35 Controls	Case-control	• HRV analyzed in stages: NREM 1–4 and REM • Time domain values computed: NN and pNN50 • Frequency domain values computed: VLF, LF, HF, and LF/HF ratio	• RR intervals and pNN50 did not change significantly from NREM to REM sleep or between PD patients and controls. • Compared to controls, PD patients had significantly lower LF values and higher HF values in both NREM and REM • LF/HF ratio remained significantly lower in PD patients than in control subjects, both in NREM and REM
Sorensen et al. (32)	Denmark	iRBD, PD	11 iRBD 14 PD with RBD 16 PD without RBD 17 Controls	Case-control	• Heart rate response (HRR) was measured instead of typical time or freq domains • HRR to arousals or Leg Movement was estimated by calculating the change in RR intervals in the ECG signal and determining the area under the curve (AUC) for the HR change (HRC) from 10 beats before to 15 beats after the onset of the events. • Heart rate response associated with arousal or leg movement from all sleep stages were analyzed.	• The heart rate response to arousals was significantly lower in both Parkinsonian groups compared with the control group and the iRBD group in N2 and REM • The heart rate response to leg movement was significantly lower in both Parkinson's groups and in the iRBD group compared with the control group. In N2 and REM
Sorensen et al. (33)	Denmark	iRBD and PD	11 iRBD 10 PD with RBD 13 PD without RBD 10 Controls	Case-control	• 5-min ECG segments taken from wakefulness, and non-REM and REM sleep • The 5-min wake was selected from the pre-sleep period and the 5-min NREM 2 was selected from the beginning of the night. • Where possible, the 5-min REM sleep was selected from the last REM period, as this tended to be the longest period of REM in most subjects. • Time domain values computed: NN, SDNN, RMSDD, NN50, and pNN50 • Frequency domain values computed: VLF, LF, HF, and LF/HF ratio	• For the iRBD patients, only the VLF component in the wakefulness stage was significantly different (lower) from the control group. • For the PD patients, SDNN, VLF, and LF were significantly lower than the control group in the wakefulness stage.
Salsone et al. (34)	Italy	RBD and PD	20 PD with RBD 20 PD without RBD	Comparative	• A circadian (24-h) HRV recording was performed in all patients (patients were independent in their activities) • The mean values of the different measures of the nighttime (from 10 p.m. to 6 a.m.) and daytime (from 9 a.m. to 10 p.m. and from 6 a.m. to 9 a.m. of the next day) HRV were calculated • Frequency domain values computed: LF and HF	• Both nocturnal LF and HF spectral power values were significantly higher in PD-RBD patients than in PD patients • PD-RBD patients LF and HF values were higher at night than during the day while no difference between nighttime and daytime values was observed in patients with PD.
Yang et al. (35)	South Korea	iRBD (RSWA)	47 iRBD 26 Controls	Case-control	• Analyzed first 5 min with stable ECG in each stage: N2, Wake, and REM • Time domain values computed: NN, SDNN, and RMSDD • Frequency domain values computed: LF and HF	• iRBD group showed reductions in SDNN, RMSSD, and HF values. • Quantified tonic RSWA was negatively correlated with LFnu values and the LF/HF ratio and postively correlated with HFnu values.

AD, Alzheimer's disease; DBP, diastolic blood pressure; ECG, electrocardiogram; H&Y, Hoehn and Yahr score; HF, high frequency; HRV, heart rate variability; iRBD, idiopathic REM sleep behavior disorder; LF, low frequency; MSA, multiple systems atrophy; N2, non-rapid eye movement (NREM) stage 2; N3, NREM stage 3; NN, normal-to-normal; nu, normalized units; PD, Parkinson's disease; PSG, polysomnography; pNN50, percent of normal-normal intervals differing >50ms from previous; PR, pulse rate; REM, rapid eye movement sleep; RMSDD, root mean square of successive differences; RR interval, interval between successive R-wave in QRS complex on ECG, RSWA, REM sleep without atonia; R_s/w_, an index of tonic heart rate decrease induced by sleep; R_bm_, the ratio of longest RR interval before body movement to shortest RR interval after body movement; SBP, systolic blood pressure; SDNN, standard deviation of normal-to-normal (NN); SAOCA, sporadic adult onset cerebellar ataxia intervals; UPDRS, Unified Parkinson's Disease Rating Scale; ULF, ultra-low frequency power; VLF, very low frequency.

As previous meta-analysis (36) has synthesized differences in HRV between patients with PD, mostly during awake studies, this narrative review aims to provide an overview of the current knowledge concerning sleep-related HRV in synucleinopathies and discuss potential contributing mechanisms. The PubMed database was searched by using the keywords “Heart rate variability” AND “sleep,” AND “alpha synucleinopathies,” OR “Parkinson's disease,” OR “Alzheimer's disease,” OR “Rapid eye movement sleep behavior disorder,” OR “multiple system atrophy” OR “dementia with Lewy Bodies.” We selected only those articles that measured HRV during sleep in synucleinopathies based on the title and abstract.

## Sleep-related heart rate variability in idiopathic REM sleep behavior disorder

RBD is characterized by REM sleep without atonia (RSWA) and dream-enactment behavior and is an early manifestation of synucleinopathies (37). RBD in individuals without a diagnosis of PD, DLB, or MSA is termed idiopathic RBD (iRBD), and termed symptomatic RBD in individuals with these diagnoses. Longitudinal cohort studies show that >80% of patients with iRBD diagnosed with a neurodegenerative disease over 12 years of follow up (3, 37, 38). Several case-control studies evaluated HRV comparing iRBD or RWSA to controls.

One case-control study showed that patients with iRBD had decreased cardiac autonomic function (35). The authors evaluated the first 5-min each of stages N2, REM, and wake recorded during video polysomnography (PSG). Compared to controls, the iRBD group showed reductions in SDNN, RMSSD, and HF values. Furthermore, quantified tonic EMG activity was inversely correlated with normalized LF values (LFnu) and LF/HF ratios and positively correlated with normalized HF values (HFnu). These results suggest that parasympathetic activity is disrupted in iRBD.

Another study (22) evaluated the ratio of R-R intervals preceding and following body movements during NREM and REM sleep in 14 patients with RBD compared to 14 matched controls. Notably, four of the RBD patients in this study had other neurodegenerative diseases (Alzheimer's disease, PD, or MSA). There was a decrease in the R-R interval ratio (reduced tachycardic response) related to body movements during REM sleep and NREM in RBD compared to controls. There were no differences between idiopathic RBD and symptomatic RBD.

One retrospective study evaluated 10 patients with iRBD compared to 10 matched controls (26). The expected increase in LF and LF/HF and the expected decrease in HF in the transition from NREM to REM sleep occurred only in controls but was absent in iRBD. Thus, the typical sympathetic predominance during REM sleep appears to be lost or decreased in iRBD.

One case-control study investigated individuals with iRWSA (21), finding no significant differences in HRV time or frequency domains during wakefulness or the first 5 min of the first N2 and last REM stages compared between 33 individuals with iRSWA and 28 controls (21). In addition, this study reported a non-significant reduction in NN during wake and NREM in individuals with iRSWA compared to controls. Taken together, case-control studies in iRBD suggest that there may be both sympathetic and parasympathetic dysfunction measurable during sleep through HRV. However, more research in larger cohorts is needed to fully understand sleep-related HRV changes in RBD.

## Sleep-related heart rate variability in idiopathic Parkinson's disease

The prevalence of ANS dysfunction in PD patients, which includes constipation, urinary dysfunction, erectile dysfunction, and orthostatic hypotension, is estimated to be between 50% and 70% (15). Because dysautonomia can occur during prodromal stages of PD, monitoring HRV parameters may aid in earlier diagnosis (15). Several case-control studies assessed sleep-related HRV parameters in both time and frequency domains in PD. The studies found that PD patients have altered cardiac autonomic function during the night or during sleep compared to controls.

Three studies evaluated 24-h ECG for HRV in PD and matched controls. One study (27) compared 13 PD and 13 controls and found significantly reduced SDNN, LF, and HF in PD compared to controls during nighttime hours. PD patients also had reduced SDNN and LF during the full 24-h period and during the day compared to controls. A larger study (23) evaluated 54 dopaminergic-naïve PD patients and 47 age-matched controls. Over 24-h, SDNN, VLF, LF, and HF were significantly lower in PD than controls. The night-time HRV was not analyzed separately, but was reported in another paper (30), showing lower night time LF and HF in PD compared to controls. This group also showed negative correlations between total and motor UPDRS and 24-h LF and VLF, but no relationship between HRV and disease duration (23). Additionally, patients with less bradykinesia had significantly higher 24-h HF. Another study evaluated 24-h HRV and included actigraphy to distinguish between time in and out of bed (28), finding that PD patients had lower HF when in bed, but higher HF when out of bed. The LF/HF ratio was lower (parasympathetic predominance) in patients than controls both in and out of bed. A limitation of these studies was the absence of EEG identification of sleep or wake.

PD patients also had altered HRV in response to body movements during REM sleep in a study that compared 26 untreated PD patients to 15 controls (22). This study found reduced tachycardia response to body movements during REM sleep in PD compared to controls.

In another study, 35 PD patients demonstrated lower sympathetic influence on HRV during both REM and NREM compared to 35 matched controls. No differences were found in time domain HRV parameters (RR interval and pN50) for REM or NREM. However, in the frequency domain, LFnu was lower and HFnu was higher in PD compared to controls, representing reduced sympathetic influence. Similarly, LF/HF was lower (parasympathetic predominance) in PD patients than controls for REM and NREM. Dopaminergic medications did not influence the HRV parameters (31). The authors attributed the findings to partial post-ganglionic noradrenergic cardiac denervation corresponding to observed blunted sympathetic responses during dream enactment behavior.

Palma et al. (16) evaluated HRV during REM and NREM (N1-N2 combined, and separately N3) sleep in 33 PD participants compared to 29 matched controls. Time and frequency domain HRV parameters were analyzed by averaging 10-min epochs across REM, N1-N2, N3, and wake. RR intervals were lower in PD compared to controls in wake, N3, and REM, with no differences in SDNN in any sleep stage. ULF was lower in N3, VLF and LF were lower during REM sleep, and HF was lower in N1-N2 in PD compared to controls. Furthermore, Unified Parkinson's Disease Rating Scale (UDPRS) part III ON and OFF scores were inversely related to VLF and LF during REM sleep. Similarly, Covassin et al. (20) found that lower HF (parasympathetic) HRV during REM sleep and higher LF/HF (sympathetic) during REM sleep were significantly correlated with disease severity in PD. These studies highlight the significance of cardiac autonomic dysfunction in PD and suggest utility in measuring disease severity.

Another study compared HRV and diffusion tensor imaging (DTI) in the medulla between 52 PD patients and 24 controls (29). PD patients did not demonstrate the expected sympathetic predominance during REM sleep, with significantly lower LF and higher HF compared to controls. In contrast to controls, there was no change from N3 to REM in either LF or HF. DTI measures in the medulla were negatively correlated with LF, HF, and LF/HF ratio in REM but not in N3, suggesting that neurodegeneration in the medulla influences loss of sympathovagal balance during REM sleep.

In addition to these case-control studies, Liu et al. (39) evaluated effects of subthalamic nucleus (STN) deep brain stimulation (DBS) on nighttime HRV in PD by comparing nights with DBS on vs. off. They found increased LF/HF ratio during the DBS-on night, suggesting that DBS may restore sympathetic regulation. However, a significant limitation of this study is that sleep and sleep stage were not confirmed during the recordings.

Thus, sleep-related HRV measures suggest alterations in sympathovagal balance and reduced sympathetic predominance in REM in people with PD. Additionally, these measures appear to be related to motor disease severity.

## Heart rate variability and multiple system atrophy

Multiple system atrophy (MSA) is a neurodegenerative disorder with glial cytoplasmic synuclein inclusions that results in autonomic dysfunction and Parkinsonism or cerebellar ataxia (19). Only two small case-control studies have evaluated HRV during sleep in MSA and another study compared HRV in MSA, PD, and controls.

One study compared seven MSA patients and seven controls, showing lower RR50, LF, and HF during sleep in MSA compared to controls (24). In controls, the LF/HF ratio was lower (parasympathetic predominance) during sleep than wake; however, this variation was absent in MSA. Another case-control sleep study showed increased HFnu in 7 MSA patients compared with 5 controls during REM and NREM 3/4 sleep (17). These findings suggest relative abnormal predominance of parasympathetic activity in MSA during REM sleep.

Brisinda et al. (19) evaluated HRV during 24-h ECG in PD, MSA, and controls showing lower LF and HF power in PD and MSA during sleep and awake activity, although PD participants had more impairment in LF/HF (parasympathetic predominance) during sleep and activity, while MSA patients had impaired LF/HF (parasympathetic predominance) only during daytime activity. However, the study is difficult to interpret because it was unclear how sleep was defined and no EEG was performed. Larger studies of sleep-related HRV are needed to explore autonomic dysfunction during sleep in MSA.

## Heart rate variability and Lewy body dementia

DLB is a synucleinopathy that manifests as progressive cognitive decline, cognitive and attentional fluctuations, visual hallucinations, RBD, and Parkinsonism. The neuronal synuclein deposits associated with DLB can affect various areas of the brain, including those that regulate the ANS. To the best of our knowledge, there have been no studies evaluating HRV during sleep in patients with DLB to date. In a study that evaluated 5-min EEG recordings of RR intervals during the day comparing DLB to Alzheimer's disease, lower values were found in DLB for most HRV parameters, including SDNN, pNN50, RMSSD, VLF, LF, HF, and total power (25). These findings suggest autonomic dysfunction in DLB and the authors proposed that HRV evaluation may be one way to distinguish between these two causes of dementia.

### Comparative studies

Cardiac autonomic dysfunction is associated with both iRBD and PD. However, whether ANS dysfunction is specific to RBD or if PD patients without RBD also demonstrate this dysfunction remains unclear.

One study (32) found attenuated heart rate responses (HRR) to both leg movements and arousals during both REM and NREM in patients with iRBD, PD-RBD, and PD-noRBD compared to controls, but no difference between PD patients with and without RBD. Sub-analysis showed that the attenuated HRR was not caused by dopaminergic medications. The most pronounced attenuation was found in PD patients, with iRBD having findings intermediate between PD and controls. These changes may be related to degeneration of cortical and subcortical regions, which are thought to be involved in modulating sleep EEG, HRR, and motor activity (40). As iRBD often precedes PD, the ANS changes may be early manifestations of brainstem neurodegeneration. HRV parameters during wakefulness and during consolidated sleep (without arousals) were also evaluated in this cohort, showing no differences in LF, HF, or LF/HF during N2 or REM between controls, iRBD, PD with RBD, and PD without RBD (33).

Another study (34) conducted 24-h ambulatory ECG recordings to elucidate whether RBD might influence circadian cardiac autonomic activity. They found increased nocturnal (10 pm to 6 am) LF and HF in PD-RBD compared to patients with PD without RBD. Further, there was no difference between daytime and nighttime LF and HF in PD without RBD, but these HRV parameters were higher at night compared to daytime in PD-RBD. These findings are unexpected based on other studies finding suppression of these measures during sleep in RBD. Because EEG was not recorded in this study, it is possible that differences between the groups in terms of percentages of sleep stages and time spent awake during the night may have been responsible for these unexpected results.

These studies demonstrate the importance of identifying sleep stages when evaluating nocturnal autonomic fluctuations, although such methods have shown mixed results when evaluating the influence of RBD on ANS in PD patients. One study (33) found no frequency domain HRV differences during sleep between PD with and without RBD. However, Bugalho et al. (18) found significant attenuation of parasympathetic HRV values (HF) in all stages of sleep in RBD (iRBD and PD-RBD) compared to PD without RBD. Further, there was a blunting of the expected increase of HF in NREM. Divergent results in these studies may be related to demographics in that participants had a higher mean age in the Bugalho study. Additionally, the Bugalho study included both iRBD and PD-RBD in the RBD group and compared to PD without RBD, while the other study compared the groups to a control group. Further study is needed to definitely determine the influence of RBD on HRV in PD.

## Discussion

**Figure 1:**
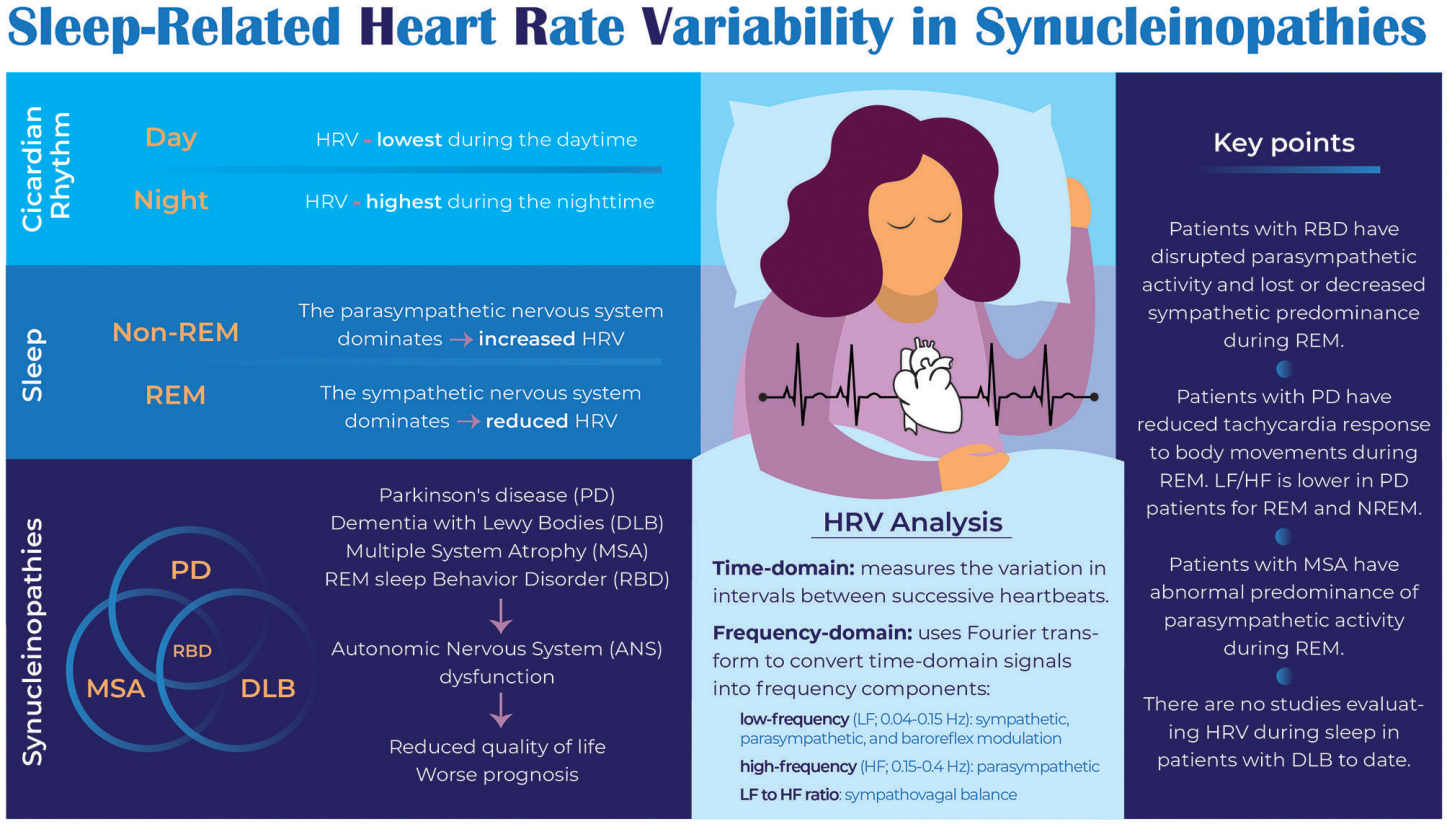
Infographic on sleep-related HRV in synucleinopathies.

This review summarizes available literature investigating sleep-related HRV in synucleinopathies, demonstrating nocturnal ANS dysfunction in these individuals. Studying HRV during sleep may provide clues as to potential biomarkers or therapeutic targets for these disorders. Figure 1 summarizes the key findings of the paper.

Many questions remain regarding the pathophysiological mechanisms of ANS dysfunction in iRBD and PD. However, neuropathological and imaging studies conducted in iRBD patients demonstrate abnormalities in several brainstem areas (41) that are important to autonomic network function of the central nervous system (CNS) (26). In PD, deposition of alpha-synuclein in both the PNS and CNS and post-ganglionic neuronal degeneration may lead to autonomic dysfunction (15). According to the Braak hypothesis (42), since iRBD often precedes PD clinical manifestations (8), the change in autonomic function may be an early manifestation of neurodegeneration in the brainstem.

There are also studies assessing whether ANS dysfunction is predominantly related to RBD or also influenced by PD pathology. There was a marked reduction in cardiac 123I-labeled meta-iodobenzylguanidine scintigraphy (a physiological norepinephrine analog used to assess sympathetic autonomic function) in patients with iRBD in the same range as PD (43, 44). However, another study found that the degree of MIBG dysfunction in iRBD was intermediately reduced between controls and patients with PD (33, 45). Using HRV as a marker, one study (34) found an increase in nocturnal sympathetic and parasympathetic activity in PD-RBD patients compared to PD-noRBD patients. Because the study lacked EEG sleep staging, it is difficult to speculate whether or how RBD may increase nocturnal ANS drive. ANS alterations could result from dream enactment behavior or microawakenings, or from cardiac nonadrenergic fiber involvement. Another proposed mechanism is spread of alpha-synuclein to pontomedullary structures, particularly the sublaterodorsal nucleus and subcoerulus complex (46). Additionally, the dorsal motor nucleus of the vagus, the nucleus solitarius, and the rostral ventrolateral medulla may be affected during the early disease stages, causing parasympathetic nervous system dysfunction (46). Future prospective studies are needed to clarify these conflicting results.

A few small studies suggest that ANS dysfunction occurs during sleep in MSA. Thus far, no studies have evaluated sleep-related HRV in DLB. More studies are needed to determine whether HRV may serve as an effective biomarker for earlier detection of MSA or DLB and provide further insight into the autonomic imbalance present in these disorders.

Although several case-control and/or retrospective studies of nocturnal HRV are available, much remains to be investigated. For example, longitudinal studies should examine how changes in sleep-related HRV impact health outcomes such as cognitive and motor decline in synuclein disorders. Additional areas of study should include wearable technology and sophisticated analytical methods. Moreover, HRV data from diverse populations should be collected to allow better understanding of normative values during sleep as well as the effects of age, sex, race/ethnicity, and comorbidities on HRV outcomes. Future research may identify clinical applications of HRV during sleep, such as its utility as a biomarker for various sleep or ANS disorders in synucleinopathies. In addition, interventional studies examining effects of sleep therapies such as cognitive-behavioral therapy, exercise, or pharmacological treatments on HRV during sleep could broaden treatment options for ANS dysfunction in neurodegenerative disorders. This review explored the nuances of HRV during sleep in synucleinopathies. Because this review is narrative, we did not follow PRISMA guidelines, and synthesizing evidence involves inherent subjectivity and potential for bias.

## Conclusion

HRV during sleep is a non-invasive measure that can inform our understanding of ANS dysfunction in synuclein disorders. Available research in iRBD, PD, and MSA demonstrate alterations in parasympathetic and sympathetic function as well as sympathovagal balance. Additional work is needed to determine if sleep-related HRV can be used as a biomarker of early disease identification, prognosis, or progression and if guided therapies targeting ANS dysfunction in sleep could improve patient outcomes and quality of life.

## Author contributions

AM: Conceptualization, Methodology, Writing – original draft, Writing – review & editing. EG: Data curation, Methodology, Writing – review & editing. TN: Data curation, Methodology, Writing – review & editing. YS: Data curation, Methodology, Writing – review & editing. CC: Visualization, Writing – review & editing. AA: Conceptualization, Methodology, Supervision, Writing – review & editing.
